# The U94 Gene of Human Herpesvirus 6: A Narrative Review of Its Role and Potential Functions

**DOI:** 10.3390/cells9122608

**Published:** 2020-12-04

**Authors:** Elisabetta Caselli, Maria D’Accolti, Francesca Caccuri, Irene Soffritti, Valentina Gentili, Daria Bortolotti, Antonella Rotola, Enzo Cassai, Simona Fiorentini, Alberto Zani, Arnaldo Caruso, Roberta Rizzo, Dario Di Luca

**Affiliations:** 1 Section of Microbiology, Department of Chemical and Pharmaceutical Sciences and LTTA, University of Ferrara, 44121 Ferrara, Italy; maria.daccolti@unife.it (M.D.); irene.soffritti@unife.it (I.S.); valentina.gentili@unife.it (V.G.); daria.bortolotti@unife.it (D.B.); antonella.rotola@unife.it (A.R.); enzo.cassai@unife.it (E.C.); roberta.rizzo@unife.it (R.R.); 2 Department of Molecular and Translational Medicine, University of Brescia, 25123 Brescia, Italy; francesca.caccuri@unibs.it (F.C.); simona.fiorentini@unibs.it (S.F.); a.zani033@unibs.it (A.Z.); arnaldo.caruso@unibs.it (A.C.); 3 Section of Microbiology, Department of Medical Sciences, University of Ferrara, 44121 Ferrara, Italy; ddl@unife.it

**Keywords:** human herpesvirus 6, U94 functions, U94 exploitation

## Abstract

Human herpesvirus 6 (HHV-6) is a β-herpesvirus that is highly prevalent in the human population. HHV-6 comprises two recognized species (HHV-6A and HHV-6B). Despite different cell tropism and disease association, HHV-6A/B show high genome homology and harbor the conserved U94 gene, which is limited to HHV-6 and absent in all the other human herpesviruses. U94 has key functions in the virus life cycle and associated diseases, having demonstrated or putative roles in virus replication, integration, and reactivation. During natural infection, U94 elicits an immune response, and the prevalence and extent of the anti-U94 response are associated with specific diseases. Notably, U94 can entirely reproduce some virus effects at the cell level, including inhibition of cell migration, induction of cytokines and HLA-G expression, and angiogenesis inhibition, supporting a direct U94 role in the development of HHV-6-associated diseases. Moreover, specific U94 properties, such as the ability to modulate angiogenesis pathways, have been exploited to counteract cancer development. Here, we review the information available on this key HHV-6 gene, highlighting its potential uses.

## 1. Introduction

Human herpesvirus 6 (HHV-6) is the common name for two now-distinct viruses, HHV-6A and HHV-6B. Originally isolated in 1986 from blood lymphocytes of adult subjects affected by lymphoproliferative disorders, it was for that reason initially named human B-lymphotropic virus [1]. Soon afterward, however, the virus was found in CD4+ T-lymphocytes, which represent the preferential target cell of the virus [2], and the species 6B was later recognized to be causally associated with the onset of *roseola infantum* (also denominated *exanthema subitum* or sixth disease), a febrile illness in infants.

Long considered a unique virus that is distinct in two variants, HHV-6A and 6B were recently recognized as different virus species [3] based on their different biological properties, cell tropism, epidemiology, and disease associations, despite their high degree of genome homology. HHV-6 is the only human herpesvirus, among those sequenced, to contain the U94 gene, which is highly conserved in both virus species [4]. The U94 gene product has been recognized to have a crucial role in virus replication and peculiar effects in infected cells. Here, we summarize the current knowledge on this virus protein and its potential uses to modulate specific cell functions.

## 2. HHV-6 Epidemiology and Disease Association

HHV-6 belongs to the *β-Herpesvirinae* subfamily of the *Herpesviridae* family, where it was included on the basis of its genomic similarities with human cytomegalovirus (HCMV) [5]. Along with HHV-7, it constitutes the *Roseolovirus* genus, and its infection is widespread worldwide [4]. The two species, HHV-6A and HHV-6B, share high sequence identity (ranging from 75% to 95% depending on the considered genome region) but differ in genome sequence [6,7,8] as well as cell tropism, growth properties, antigenic properties, epidemiology, and disease association [9,10,11,12].

The two viral species use different cell receptors: HHV-6A uses CD46 [13], expressed on all nucleated cells, whereas HHV-6B uses CD134, expressed on T-lymphocytes [14]. Both HHV-6 species replicate most efficiently in vitro in activated primary T-cells as well as in continuous T-cell lines. However, although originally classified as lymphotropic herpesviruses, both HHV-6A and HHV-6B species have a considerably broader cell tropism as they can infect not only T- and B-lymphocytes but also macrophages [15], natural killer (NK) cells [16], vascular and lymphatic endothelial cells [17,18,19], epithelial cells of the thyroid [20,21], salivary glands [4,22], endometrium [23], skin [24], fibroblasts [25], and olygodendrocytes and neurons [26]. In vivo, HHV-6A/B can be detected from a broad range of cells and tissues, including lymph nodes, peripheral blood mononuclear cells (PBMCs), renal tubular cells, salivary glands, thyroid, skin, and the central nervous system [15,26,27,28,29,30,31]. A retrospective investigation on the virus species detected in the mentioned studies, based on the viral strains used as described in the methods of the studies, revealed that the 6A species (strains GS, U1102-like) was mostly detected in the central nervous system [26], although HHV-6B was also reported in multiple sclerosis plaques [31]. Both 6A (AJ strain) and 6B (Z29 strain) species were detected in saliva [28,30], whereas 6B was mainly found in PBMCs and kidney cells [28,29]. HHV-6 has also been identified in astrocytes from gliomas, initially suggesting a potential role in tumorigenesis, especially for the 6A species, which has been found in 72% of HHV-6-positive pediatric tumors [32,33]. However, recent studies based on the application of the International Agency for Research on Cancer (IARC) criteria showed insufficient evidence for considering HHV-6 an oncogenic virus in lymphomas and brain cancer [34].

Like all human herpesviruses, HHV-6 causes both productive and latent infections. The vast majority of documented primary infections and reactivation events are linked to HHV-6B, which usually infects children within the first two years of life, causing *roseola infantum*, a disease characterized by fever, diarrhea, and mild skin rash [2,35]. Complications of this benign pathology can include febrile seizures and epilepsy. By contrast, primary infection by HHV-6B in adults is rare and can be associated with a mononucleosis-like syndrome, with prolonged lymphadenopathy [36]. Less is known about the epidemiology or clinical implications of HHV-6A, which has long been considered not as prevalent compared to HHV-6B and whose causal role in primary infection and subsequent disease is still poorly understood [37]. However, recent findings suggest that HHV-6A is actually quite common, based on expensive serological screening using a method that is able to discriminate between the two species [38]. 

After primary infection, HHV-6 establishes a life-long latent infection in the host, from which it may reactivate, especially during immune dysregulation [39]. Several diseases have been associated with HHV-6A/B reactivation in adults, although the causal correlations are still unproven. Most studies on pathogenic association do not specify the HHV-6 virus species, which, however, can be inferred by the reference strains used in the methods. HHV-6A/6B-associated pathologies include neurological diseases in immunocompromised subjects, such as encephalitis, seizures, ataxia, and mild dementia, with a higher prevalence of HHV-6A in cognitive dysfunctions and of HHV-6B in encephalitis and seizures [40,41,42,43,44,45]. Both virus species have further been correlated to multiple sclerosis (both HHV-6A and HHV-6B; the latter is predominantly found in PBMCs of patients) [46,47,48,49,50], systemic sclerosis (HHV-6A) and connective tissue diseases (both HHV-6A/6B) [24,51,52,53], Hashimoto’s thyroiditis (HHV-6A) [20,54], female infertility (HHV-6A) [23,55], fulminant hepatic failure (HHV-6A/6B), chronic fatigue syndrome (HHV-6A) [47,56], and neoplasia, myocarditis, drug reaction with eosinophilia, and systemic symptoms (HHV-6A/6B) [57,58]. A possible role of HHV-6A infection has also been recently suggested in Alzheimer’s disease [59,60]. Reactivation of HHV-6B has been recently documented in a COVID-19 patient [61], which may be correlated with the increased expression of the CD134 HHV-6B receptor and the inflammatory cytokine IL-6 [62]. Consistent with this observation, HHV-6-associated diseases such as *Pityriasis rosea* and Kawasaki’s disease increased around ten-fold during the COVID-19 pandemic, compared to previous periods [63].

## 3. HHV-6 Genome and U94 ORF

**Figure 1:**
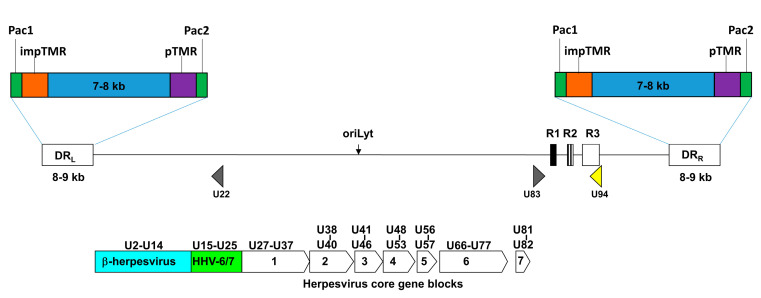
Schematic representation of the human herpesvirus 6 (HHV-6) genome. The U region, containing core genes and β-herpesvirus genes flanked by direct repeats (DRs), is shown. Genes unique to human *Roseoloviruses* or HHV-6 are outside this block. The DRs are flanked by the packaging sequences (Pac 1 and Pac 2) and two telomeric repeats (pTMR and impTMR). The origin of replication (oriLyt) and the U94 ORF location are evidenced.

The HHV-6 genome consists of a double-stranded linear DNA segment around 160 Kb long, with slight differences between 6A (about 156 Kb in size for the GS strain) and 6B (about 161 Kb long for the Z29 strain) [64]. Virus genome was completely sequenced in 1995 [65] and contains over 100 open reading frames (ORFs), mostly located in the unique region (U), which is surrounded by 8–9 Kb direct repeats (DR, DR_L_ left, and DR_R_ right), containing 9 ORFs, elements needed for viral DNA packaging (pac1 and pac2), and human telomere-like (TAACCC) simple repeats, including perfect and imperfect telomeric repeats (TMRs). These sequences are about 0.35 Kb long but imperfect TMRs are longer than the perfect TMR and both show length variation between strains. The lengths also appear to be different between free virus and iciHHV-6A/B [65,66,67] (Figure 1). The U region contains the core genes conserved in all the herpesviruses, genes that are only present in β-herpesviruses, and genes uniquely present in HHV-6A/B, including U83 and U94 [68].

Indeed, recent genome-wide analyses using RNA sequencing (RNA-seq) and ribosome profiling revealed more complex transcriptomes [64], suggesting that the current annotations of the HHV-6 genome are likely incomplete. In particular, novel conserved translation patterns and noncoding RNAs were identified in HHV-6A and -6B (some of which were also conserved in HCMV), with possible central functions in all β-herpesviruses [64]. The overall nucleotide sequence identity between HHV-6A and HHV-6B is around 90%, ranging from 70% to 95% in the more conserved core genes.

The most divergent regions are DRs and the right end of the U region, spanning ORFs U86 to U100 [38]. In particular, the immediate-early 1 (IE1) proteins of HHV-6A and HHV-6B, encoded by the U90–U89 genes show only 62% homology [69] and has been consistently used to develop multiplex serological assays that are able to distinguish antibodies directed against the two virus species [38]. These regions have 85% and 72% nucleotide sequence identity, respectively [70]. The most variable genes include the immediate-early 1 (IE1) gene (U90–U89), some regions of the glycoprotein B (U39) and glycoprotein H (U48) genes, and U94, which is indeed utilized to distinguish between 6A and 6B species [12,71,72].

The U94 gene has no counterparts in any of the other known human herpesviruses, even in the closely related β-herpesviruses HHV-7 and HCMV, and is located in the antisense strand of the virus genome [65,70]. Among sequenced herpesviruses, only rat cytomegalovirus (RCMV) and bat *Miniopterus schreibersii* β-herpesvirus (MsHV) encode a U94/rep homolog [73,74], although in RCMV, the U94 sequence is sometimes lacking [75]. Despite strain differences, similar genomic location and orientation suggest that the U94 gene may derive from a parvovirus progenitor integrated into a β-herpesvirus genome, where it was subsequently maintained [76].

The HHV-6 U94 gene is a spliced gene encoding a 490-aminoacid protein that is highly conserved between species, as the difference consists of 10 amino acid residues [72]. It was initially observed to encode a product homologous to the adeno-associated virus type 2 *rep* gene product (RepAAV-2), which encodes four overlapping proteins required for AAV-2 DNA replication and modulation of the expression of homologous and heterologous genes [77,78]. The RepAAV-2 product has DNA-binding, site- and strand-specific endonuclease, and helicase and ATP-ase activities [79,80]. It is necessary for AAV-2 integration [81,82], inhibits transcription of other viruses such as HIV-1 [83], and represses the expression of cell oncogenes [84]. 

U94 was shown to share 24% amino acid identity with RepAAV-2 and, consistent with this, to be able to restore the replication in Rep-deficient AAV-2 mutants [83], suggesting similar regulatory functions in the viral life cycle. Additionally, U94 was reported to bind the TATA-binding site of human transcriptional factors [85] and to suppress human *ras* oncogene expression and HIV-1 transcription [86]. The HHV-6A/6B U94 gene product further possesses single-strand and double-strand DNA-binding activity, with a preference for TTAGGG repeats, as present in human telomeres [87,88]. In addition, U94 has ATPase, helicase, and exonuclease activity in vitro [89]. 

## 4. U94 and Virus Cycle

The expression of U94 is observed during the whole life cycle of the HHV-6 virus, both in the productive/lytic and latent phases of infection. During the productive infection, the U94 gene is expressed as an immediate-early (IE) α gene before the expression of early (β) and late (γ) genes [90]. In in-vitro infected cells, the U94 transcript is expressed at low levels during lytic infection, suggesting that small amounts of U94 protein are required during the productive replication of the virus [72]. Several in vitro studies have shown the ability of U94 to bind and modulate the expression of DNA, supporting its role in the regulation and replication of virus gene expression and in the maintenance of virus latency [87]. The regulatory role of the U94 gene product was confirmed by transfecting cells with the full-length U94 gene cloned in expression plasmids [72,83,91,92] and by treating cells directly with the recombinant gene products, either in the form of fusion proteins [85] or as recombinant purified U94 protein [87]. The U94 protein inhibits the replication of HHV-6A/B, HHV-7, and HCMV in infected cells, with a dose-dependent effect, whereas it has no effect on α- and γ-herpesviruses, suggesting an action specifically directed toward the β subfamily [87]. Interestingly, the exogenously provided U94 protein accumulates in the treated cells at the nuclear level, according to its possible role in DNA replication and expression [87]. Once internalized, it inhibits the virus replication by blocking the virus cycle before genome replication (during IE/E gene expression) and not at virus entry level. The active domain appears to be harbored in the N-terminal region of the protein and to be conformational-dependent, as the heat-denatured protein and C-terminal region are inactive [87].

U94 transcripts are also found in-vivo in latently infected cells such as freshly isolated PBMCs from healthy donors, in the absence of other viral mRNAs [91,93], and have therefore been indicated as a useful marker of virus latent infection. After primary infection, in fact, like other herpesviruses, HHV-6 establishes a latent infection in the host, persisting lifelong in peripheral blood mononuclear cells (PBMCs) and in some somatic cells [15,40,94]. During the latent phase of infection, only a small subset of viral genes is expressed and U94 is one of those genes, together with four transcripts of the IE1/IE2 region (U90–U89 and U90–U86/87) [95]. U94 is considered not only a marker of latency but also a gene whose expression is important for latency maintenance. In fact, as already mentioned, T-cells stably expressing U94 are not permissive for HHV-6 lytic infection [91], and its expression inhibits HHV-6 and β-herpesvirus replication [87], thus supporting a specific role of U94 in the regulation of virus production. Overall, the U94 functions are strongly suggestive of a role in the tight control of virus replication, possibly favoring the selection of less virulent strains to favor long-term survival in the host.

## 5. U94 and Virus Integration

**Figure 2:**
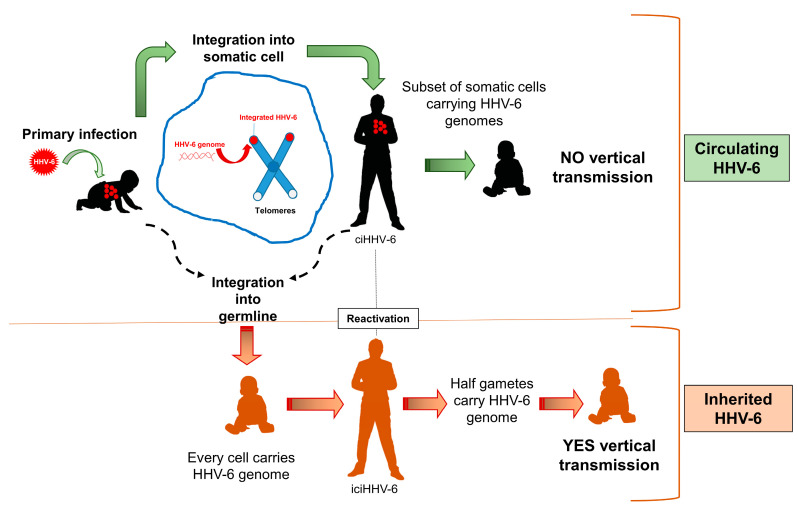
HHV-6 integration. Upper panel: the virus can integrate into the chromosomal telomeres of somatic cells during primary infection to maintain its genome in the host for life (ciHHV-6). Lower panel: HHV-6 can integrate into germ cells, resulting in the virus genome being carried in each cell of the host (iciHHV-6) [68]. The chromosome is schematically represented as an X-like structure; although not depicted, the virus genome is present in both sister chromatids.

Most herpesviruses maintain their latent genome in an episomal circular form, but HHV-6A/B can integrate their genomes into the chromosomal telomeres and no episomes are observed [15,78,96]. The integration usually occurs in a small proportion of somatic cells, including monocytes, macrophages, T-cells, or bone marrow progenitor cells [15,97,98,99]. Recent studies have shown that, when integrated, the HHV-6A genome is silent, suggesting that during latency, some epigenetic cell factors are involved in transcription silencing [100]. This allows the virus to persist in the host lifelong and sporadically reactivate. However, in addition to integration in somatic cells, HHV-6A/B have also been detected as integrated into the chromosomes of germ cells [101], suggesting that for these viruses, integration may represent more than a sporadic event (Figure 2).

The observation of a full-length integrated HHV-6 genome was first reported in the DNA of PBMCs [96,97], and since then, such a condition has been detected in about 1% of the human population, which corresponds to nearly 70 million individuals, considering that HHV-6 has a prevalence close to 100% in the world population. HHV-6 integration determines an inherited condition in newborns, where each cell carries a copy of the virus genome (inherited chromosomally integrated HHV-6 (iciHHV-6)) [101]. iciHHV-6 individuals transmit the virus according to Mendelian laws. This entry in the germ line was also observed for another herpesvirus that is closely related to HHV-6A/B, the *Tarsius syrichta Roseolovirus* 1 [102]. In the human genome, independently of the chromosome, virus integration occurs in the telomere region of the chromosome, as observed in latently infected cells [101,103]. It remains unclear whether the integration in the germ cells occurs during primary infection or reactivation. Virus integration has been indicated as a predisposing factor for angina pectoris and pre-eclampsia in pregnant women [104,105], and gene expression and virus reactivation has been detected [106,107,108]; however, the pathogenic role of the integrated virus is still undefined.

**Figure 3:**
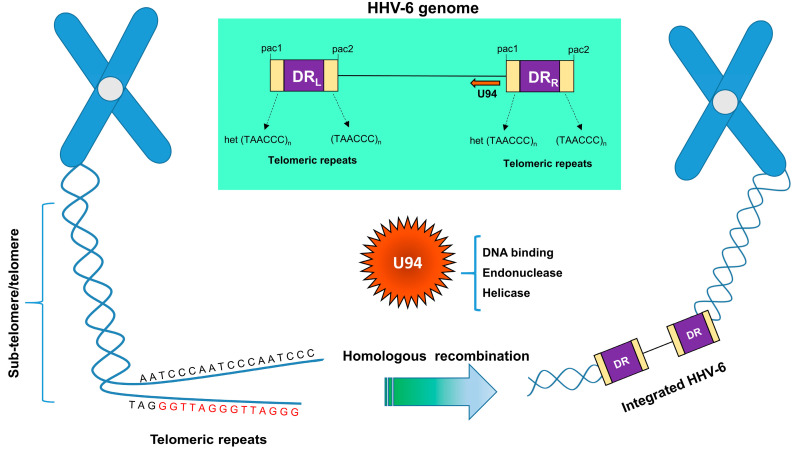
Proposed model for U94 contributions in the integration of the HHV-6 genome into the subtelomeric and/or telomeric region of human chromosomes. Chromosomal telomeric sequence is indicated. Viral direct repeats, left and right (DR_L_ and DR_R_), with pac regions containing the telomeric repeats, are also indicated. The chromosome is schematically represented as an X-like structure; although not depicted, the virus genome is present in both sister chromatids.

The mechanisms allowing HHV-6 integration are not yet elucidated, but it is likely that the TAACCC motifs present in the genome regions containing DR and packaging (pac) sequences are involved due to their homology with human telomeric repeat sequences [109,110]. Consistent with this, all HHV-6A/B integration sites have been identified in telomeric regions. The terminal PAC2 in DRR and terminal PAC1 in DRL are absent from integrated copies of the viral genome, and these data support the recombination-based mechanism of integration [111,112]. However, the presence of telomeric sequences per se is not sufficient to induce genome integration, as HHV-7, for example, harbors such sequences, but no integration has been reported so far [113]. In contrast, U94 is limited to HHV-6A/B, with respect to other even closer herpesviruses, and, due to its properties, has been hypothesized to be involved in the integration process (Figure 3). Analogous to AAV-2 Rep protein, it has DNA-binding and helicase and endonuclease activities, suggesting it might be involved in the steps needed for integration. Moreover, U94 can inhibit virus replication [87,91], supporting its role in the establishment of latency or, alternatively, virus integration. 

Interestingly, U94 transcripts were detected in the majority (54.5%) of PBMC derived from 11 individuals with iciHHV-6 [106], supporting a role for U94 during integration. Of note, while U94 mRNA is commonly detected during latency, it was found less frequently in iciHHV-6 PBMC, which might lead to the hypothesis of different mechanisms underlying latency and inherited chromosomal integration conditions. A possible model of U94 action is depicted in Figure 3.

However, the role of U94 in integration is yet unproven due to the lack of reverse-genetic systems and efficient integration assays able to quantify virus integration; more studies are needed to support the role of U94 in HHV-6A/B integration, especially in light of a recent report suggesting that U94 may be dispensable for virus integration [114]. A mutant HHV-6A, obtained by deleting the entire U94 gene (ΔU94) in a bacterial artificial chromosome (BAC), maintained the ability to integrate into cell DNA, at least in the cell lines used in the study, indicating that the virus may use more pathways to ensure its integration in the host cell genome. Viral factors, including U41 and U70, are hypothesized to participate in the mechanisms, together with cell factors, and are targets for future studies [68,114,115]. Moreover, the immediate-early protein 1 (IE-1) of HHV-6A/B was recently shown to target the cell promyelocytic leukemia protein (PML), whose knockdown strongly impairs HHV-6A/B integration [116]. Since PML has a role in DNA repair, recombination, and telomere maintenance, this observation supports its involvement in the virus integration process. Intriguingly, recent data have reported that iciHHV-6 can modulate the expression of cell genes in the host cell, particularly genes encoding immunoglobulins [117], suggesting a possible association with induction of immune deficiencies. However, as to the role of U94, further studies are needed, particularly in primary cells or in ex vivo cells, to elucidate the mechanisms underlying the regulation and maintenance of virus integration.

## 6. U94 and Immunity

Despite its nonconstitutive function, during the natural infection by HHV-6, an immune response is elicited against the virus U94 gene product, both at the humoral and cellular levels. The onset of antibodies specifically directed against the U94 protein can be observed during the seroconversion in acutely infected subjects [49] and in about 40% of the adult general population, with an antibody titer that is generally low (mean titer 1:130, range 50–240) [24,49]. Interestingly, however, a significantly higher prevalence and titer of anti-U94 antibodies have been detected in diverse autoimmune diseases, including multiple sclerosis [49], Hashimoto’s thyroiditis [20], and systemic sclerosis [24]. In particular, despite the unvaried prevalence and titer of antibodies directed against the virion proteins, which are present in over 95% of the world’s human population, the elicited immune response against the U94 regulatory protein appears increased in individuals displaying HHV-6A/B-associated autoimmune pathologies. 

In multiple sclerosis patients, >80% had anti-U94 IgG, with a mean titer of 1:515 (range 200–1200) [49]. Similarly, all (100%) of the surveyed systemic sclerosis and Hashimoto’s thyroiditis patients resulted positive for the presence of circulating anti-U94 IgG, compared to controls (respectively, 46.7% and 60% in the different control populations of the reported studies) [24,118]. Antibody titer was also significantly increased in patients affected by such autoimmune diseases, with mean values corresponding to 3- to 5-fold of those detected in controls [24,118]. Since the U94 gene is expressed both during the latent and productive phases of infection, leading to an uninterrupted stimulation of the immune system, these results suggest that in such patients, there could be higher exposure to the antigen compared to healthy controls. On the other hand, the increased prevalence and titer of anti-U94 antibodies suggest variations in U94 production or frequent virus reactivations (i.e., switches from latency to active replication) that might lead to increased sensitization to the antigen.

These observations further suggest that immune-dysregulated individuals may not properly control the infection/reactivation of HHV-6A/B, allowing multiple virus reactivations and, hence, their possible pathological consequences. Moreover, the measurement of humoral and/or cellular response directed against the U94 antigen might be utilized as a risk marker for the development of HHV-6-associated diseases by contrast with anti-HHV-6 responses, which are almost unaltered in patients and controls due to the high prevalence of the virus in the human population.

Interestingly, previous studies by our group also evidenced that U94 can also elicit a cellular immune response, showing a prominent reactivity of CD4+ and CD8+ T-cells toward the antigen, particularly the subset of CD4+ cells secreting both IFNγ and IL-2 [20], which is suggestive of a persistent immune response to the HHV-6 antigen. In parallel, an impairment of the NK response was observed when using peripheral NK cells obtained from systemic sclerosis patients in activation assays against the U94 antigen [24]. This is consistent with what has been observed in multiple sclerosis patients, where an impairment of innate response against the virus was reported [50]. Intriguingly, the expression and release of the soluble form of the nonclassical human leukocyte antigen G (HLA-G) is known to have a tolerogenic effect, for example, during the establishment of pregnancy to allow embryo implantation [119]. HHV-6A/B have been consistently reported to induce the expression of HLA-G in mesothelial cells, leading to impairment of NK functions against infected cells [120]. Importantly, the HLA-G induction is mediated by U94 since the effect is totally reproduced by the protein alone in the absence of virus infection, as demonstrated in human umbilical vein endothelial cells (HUVECs) [121]. Despite its ability to bind to the DNA sequence, U94 is not inducing HLA-G expression by binding and activating the HLA-G promoter; instead, it induces the expression of the ATF3 transcriptional factor, which, in turn, activates HLA-G expression and release [121]. The induction of HLA-G expression and release may correlate with the inhibition of the antiviral response and be part of the escape mechanisms that the virus uses to protect itself from the immune response.

## 7. U94 Effects in Cells and Tissues

Based on different studies, at least some of the pathogenic effects induced by the virus may be directly correlated with the expression of the U94 product. For example, after many years of somehow controversial results on the association between HHV-6 infection and multiple sclerosis, it has been recently shown that U94 might have a role in the demyelination process since it inhibits the migration of oligodendrocyte progenitor cells (hOPCs) [122]. This effect was also confirmed in vivo in a mouse model of myelin loss, where impaired migration of U94-expressing cells was demonstrated [122].

Of note, the possible role of U94 in host cell function had been already postulated in previous studies following HHV-6 isolation from cardiovascular tissues in vivo [123]. This first observation led to the hypothesis of the role of HHV-6 in cardiovascular diseases, similar to what is recognized for HCMV; the ability of HHV-6 to infect the endothelial cells in vitro, causing an increased release of proinflammatory cytokines, supported that hypothesis [18,19]. The most prominent effect of virus infection in such cell types, however, was an almost total impairment of the neo-angiogenic properties, correlated to the expression of U94 [17]. Studies performed in vascular and lymphatic endothelial cells showed that the cells transfected with the U94 gene or treated with the recombinant purified U94 protein lose the ability to form capillary-like structures in vitro [17]. As also observed in T-cells, once internalized by endothelial cells, the U94 protein rapidly reaches the nucleus of endothelial cells, where it induces its effects, finally impairing angiogenesis. The mechanism was at least partially elucidated, showing that U94 induces the production of soluble HLA-G via ATF3 activation [121], finally allowing HLA-G to work its antiangiogenic activity [124]. 

Of note, U94 not only inhibited capillary formation in vitro but also affected endothelial cell migration and blocked angiogenesis in rat aortic rings, suggesting possible useful applications where antiangiogenetic functions are needed, as, for example, in tumors. Toward this hypothesis, previous studies have shown that NIH3T3 cells stably expressing U94 suppressed transformation by the oncogene *ras* [125] and that U94 expression inhibited tumorigenesis of the prostate cancer PC3 cell line [126].

Based on its potent antiangiogenic and antimigration activity in endothelial cells, the biological impact of U94 was recently tested in a human breast cancer cell line (MDA-MB-231), showing that it can downmodulate the expression of *Src* and inhibit cancer cell motility, invasion, and anchorage-independent growth [127]. In detail, U94 expression obtained by an HSV-1-based amplicon reversibly arrested tumor cell growth in vitro and strongly decreased the migratory activity of cells, as measured by wound healing assays, which is a required feature for tumor dissemination and metastasis. U94 significantly inhibited (60%) the ability to form colonies of MDA-MB-231 cells, strongly decreased *Src* expression and its downstream signaling cascade, and induced a partial mesenchymal-to-epithelial transition in transduced cells, with a strong downregulation of TWIST (a recognized tumor-promoting invasion), N-cadherin, Snail1, and MMP2. In addition, U94 expression caused a dramatic decrease in tumor growth in vivo in NOD/SCID mice [125]. Interestingly, strong impaired angiogenesis was observed in xenografted tumors, supporting the hypothesis that the mechanism of action is linked to the ability of U94 to inhibit this crucial step of tumor development. These findings highlight the capability of U94 to render the endothelial cells insensitive to proangiogenic stimuli, including VEGF [17], and support its potential use as an antitumor molecule. Consistently, U94 has been shown to inhibit proliferation, invasion, and angiogenesis (also in glioma) [128], downregulating proangiogenic factors and MMPs, thus confirming, in this tumor type as well, that it might a potential target for therapeutic intervention. In addition, U94 was recently shown to inhibit DNA damage repair mechanisms and induce apoptosis in triple-negative breast cancer cells [129], which account for about 15% of breast cancers. Interestingly, U94 altered the expression of several proteins involved in intrinsic apoptosis, such as Bcl-2, Bad, caspase-3, caspase-9, and PARP, and inhibited the cholesterol biosynthesis pathway. Notably, U94 acted synergistically with DNA-damaging drugs, leading to efficient tumor cell death and opening interesting perspectives for the use of U94 or its derivatives to realize new anticancer molecules.

## 8. Conclusions

Recent advances have started to unravel the role of U94 and its possible effects at cell and tissue levels. In fact, originally investigated for its particular presence and role in HHV-6A/B, the U94 gene product has indeed revealed a plethora of actions that are even unrelated to the virus cycle. Reported findings on these aspects regarding the putative actions of HHV-6 U94 are summarized in Table 1. 

As to the viral life cycle, reported U94 functions include virus latency, integration, and reactivation; however, more information would be needed to precisely define its role and mechanism of action. Large-scale population studies, as well as systemic monitoring of iciHHV-6, could provide conclusive answers on the biological mechanisms involved in U94 action. The virus reactivates frequently in latently infected adults, as shown by the isolation of the virus from saliva, and virus reactivation can also be observed in iciHHV-6 individuals. Multiple studies have associated virus reactivation with several clinical conditions, but the mechanisms of HHV-6 reactivation and the role of U94 in these processes are still poorly elucidated. Of note, overexpression of U94 inhibits β-herpesvirus replication, including HCMV, HHV-7 (strain CZ), HHV-6A (strain U1102), and HHV-6B (strain Z29) [87], thus highlighting a potential role of U94 in developing new therapeutic targets to prevent clinical complications associated with β-herpesvirus reactivation. Moreover, based on the observations that U94 inhibits HIV-1 LTR activity [86], a broader spectrum of U94 inhibitory activity towards different types of viruses may also be hypothesized, which deserves further investigation.

At the immune response level, the production of a purified recombinant U94 protein allowed the recognition of a specific anti-U94 immune response and opened the way to studies regarding the potential association between HHV-6 infection and disease. In fact, contrary to what was observed in an anti-HHV-6 response, which is generally unmodified in specific subpopulations because of high virus prevalence in the human population, a differential response toward U94 was observed in specific patients affected by some autoimmune diseases (multiple sclerosis, systemic sclerosis, and Hashimoto’s thyroiditis). Thus, the quantification of anti-U94 humoral and/or cellular responses may be used as a marker for an association between the disease and HHV-6 infection, as well as to monitor the effectiveness of eventual antiviral therapies.

Finally, but perhaps first as to its importance in human biology, the U94 protein has been shown to possess key biological properties, some of which are directly correlated with HHV-6 pathogenesis (such as U94 action in the demyelination process). Among them, the ability of U94 to modulate the angiogenesis process, likely related to eventual vascular damage during natural virus infection, appears to have great potential as a therapeutic molecule due to the key role of neovascularization in tumor onset and progression. The results obtained by using this virus protein in vitro and in vivo are intriguing and promising and could open the way to new approaches in anticancer therapy. Based on the reports identifying the active domain of U94 in the N-terminal portion of the protein, it might be important to dissect the protein and identify the minimum active peptide toward the optimization of the anticancer effect of U94. A further interesting aspect is the inhibition of cholesterol synthesis, another pathway that deserves further investigation. Transcriptome and proteome approaches may be profitably used toward an understanding of the molecular pathways influenced by U94, possibly contributing to identifying new U94 action networks.

In conclusion, the overall picture about U94 functions is still not complete, and the properties of U94 that have been uncovered in recent years have opened the way to finding the pieces of information still lacking, hopefully leading to the development of useful tools in the management of diseases of different origin. 

## 9. Patents

A patent for the use of U94 of HHV-6 and its derivatives to modulate HLA-G expression has been obtained by the authors based on the results summarized in this manuscript (Patent n° 102018000006137).

## Figures and Tables

**Table 1:** U94 gene product findings.

Field of Action	Context	Results	Condition	Reference
**Humoral Immunity**	Anti-U94 IgG	Increased	Acutely HHV-6 infected subjects	[49]
	Anti-U94 IgG	Increased prevalence/titer compared to control population	Multiple sclerosis patients (serum/plasma)	[49]
	Anti-U94 IgG	Increased prevalence/titer compared to control population	Hashimoto’s thyroiditis patients (serum/plasma)	[20]
	Anti-U94 IgG	Increased prevalence/titer compared to control population	Systemic sclerosis patients (serum/plasma)	[24]
**Cell-mediated immunity**	Anti-U94 CD4+/CD8+ T-cells	Increased number	Hashimoto’s thyroiditis patients (purified PBMCs)	[20]
**Innate immunity**	NK cells	Decreased NK activation	Systemic sclerosis patients (purified PBMCs)	[24]
**Mesothelium**	Mesothelial cells	HLA-G induction	In vitro impairment of NK response against infected cells	[120]
**Demyelination (multiple sclerosis)**	Oligodendrocytes progenitors	Inhibition of migration of hOPCs	In vitro studies; animal model (NSG mice)	[122]
**Angiogenesis**	Vascular and lymphatic endothelial cells	Inhibition of angiogenesis	In vitro studies	[17]
	Vascular endothelial cells (HUVEC)	HLA-G induction (via ATF3 activation)	In vitro studies	[121]
	Rat aortic rings	Inhibition of migration	Ex vivo studies	[17]
**Tumorigenesis**	NIH3T3 cells	Suppression of transformation by *ras* oncogene	In vitro studies	[126]
	PC3 cells	Inhibition of tumorigenesis	Animal model (athymic nude mice)	[127]
	MDA-MB-231 cells	Down-modulation of *src* oncogene; inhibition of cell motility and invasion; mesenchymal-to-epithelial transition	In vitro studies	[125]
	MDA-MB-231 cells	Down-regulation of TWIST, N-cadherin, Snail1 and MMP2	In vitro studies	[125]
	MDA-MB-231 cells	Decrease of tumor growth	Animal model (NOD/SCID mice)	
	Glioma	Down-regulation of proangiogenic factors and MMPs	Ex vivo studies (human gliomas)	[128]
	Triple-negative breast cancer (TNBC) cells	Inhibition of DMA repair mechanisms, induction of apoptosis; synergistic action with anticancer drugs	In vitro studies	[129]
